# One Health concept for strengthening public health surveillance and response through Field Epidemiology and Laboratory Training in Ghana

**Published:** 2011-12-14

**Authors:** Frederick Wurapa, Ebenezer Afari, Chima Ohuabunwo, Samuel Sackey, Christine Clerk, Simon Kwadje, Nathaniel Yebuah, Joseph Amankwa, George Amofah, Ebenezer Appiah-Denkyira

**Affiliations:** 1 School of Public Health, University of Ghana, Ghana; 2 Veterinary Services Department, Ministry of Food and Agriculture, Ghana; 3 Ministry of Health, Ghana Health Services, Accra, Ghana; 4 Morehouse School of Medicine, Atlanta GA, USA

**Keywords:** Ghana, public health, One Health, epidemics, Trainees, FELTP, applied epidemiology

## Abstract

The lack of highly trained field epidemiologists in the public health system in Ghana has been known since the 1970s when the Planning Unit was established in the Ghana Ministry of Health. When the Public Health School was started in 1994, the decision was taken to develop a 1 academic-year general MPH course. The persisting need for well-trained epidemiologists to support the public health surveillance, outbreak investigation and response system made the development of the Field Epidemiology and Laboratory Training Programme (FELTP) a national priority. The School of Public health and the Ministry of Health therefore requested the technical and financial assistance of the United States Centers for Disease Control and Prevention (CDC) in organizing the Programme. The collaboration started by organizing short courses in disease outbreak investigations and response for serving Ghana Health Service staff. The success of the short courses led to development of the FELTP. By October 2007, the new FELTP curriculum for the award of a Masters of Philosophy in Applied Epidemiology and Disease Control was approved by the Academic Board of the University of Ghana and the programme started that academic year. Since then five cohorts of 37 residents have been enrolled in the two tracks of the programme. They consist of 12 physicians, 12 veterinarians and 13 laboratory scientists. The first two cohorts of 13 residents have graduated. The third cohort of seven has submitted dissertations and is awaiting the results. The fourth cohort has started the second year of field placement while the fifth cohort has just started the first semester. The field activities of the graduates have included disease outbreak investigations and response, evaluation of disease surveillance systems at the national level and analysis of datasets on diseases at the regional level. The residents have made a total of 25 oral presentations and 39 poster presentations at various regional and global scientific conferences. The Ghana FELTP (GFELTP) has promoted the introduction of the One Health concept into FELTP. It hosted the first USAID–supported workshop in West Africa to further integrate and strengthen collaboration of the animal and human health sectors in the FETP model. GFELTP has also taken the lead in hosting the first AFENET Center for Training in Public Health Leadership and Management, through which the short course on Management for Improving Public Health Interventions was developed for AFENET member countries. The GFELTP pre-tested the Integrated Avian Influenza Outbreak and Pandemic Influenza course in preparation for introducing the materials into the curriculum of other FELTP in the network. The leadership positions to which the graduates of the program have been appointed in the human and animal Public Health Services, improvement in disease surveillance, outbreak investigation and response along with the testimony of the health authorities about their appreciation of the outputs of the graduates at various fora, is a strong indication that the GFELTP is meeting its objectives.

## Introduction

At the request of the Ghana Ministry of Health, the University of Ghana established the School of Public Health (SPH) in October 1994. This is a 1-year course in general public health which awards a Master of Public Health (MPH) degree. The SPH was one of the beneficiaries of the Rockefeller Foundation support to the network of Public Health Schools Without Walls (PHSWOW) in the Africa Region [1].

Graduates of the SPH were found to meet the expectations of the Ministry of Health, as they took up leadership roles at district level. It was however, realized that a cadre of highly-trained epidemiologists with competencies and skills in applied epidemiology and disease control was needed to manage the existing complex of public health emergencies and emerging and re-emerging diseases, such as Severe Acute Respiratory Syndrome(SARS) and Avian influenza. During the early stages of implementation of the Global Programme for the Control of Malaria, HIV/AIDS and Tuberculosis, the lack of highly trained field epidemiologists became more apparent as the demand for expert management, interpretation and use of disease surveillance data increased. Unfortunately, the MPH Programme did not make provisions for the training of this cadre of professionals. A process was initiated to establish a Field Epidemiology and Laboratory Training Program (FELTP) to address the identified need. The Ghana FELTP (GFELTP) evolved from an initial collaboration with the United States (U.S.) Centers for Disease Control and Prevention (CDC), through cooperative agreements with the SPH. Activities supported by this cooperation included organization of short courses on disease surveillance, outbreak investigations and response. More than 60 serving district health staff (frontline health workers) and MPH graduates benefited from these short courses over a three-year (2003-2005) period [2]. Parts of the short course materials were later incorporated into the MPH curriculum of the School of Public Health. When, in 2005, the decision was taken to start an FELTP, the task of designing the curriculum was spearheaded by the faculty under the guidance of staff of CDC including staff from the Sustainable Management Systems Development Program (SMDP) [3].

The FELTP curriculum was adapted from CDC's core FETP curriculum [3]. GFELTP graduates receive a Master of Philosophy (MPhil) in Applied Epidemiology and Disease Control upon completing all university requirements. In addition, graduates receive certificate of competency in field epidemiology. The program was approved by the University Academic Board and the National Accreditation Board in 2007. The program started with an initial cohort of three physicians, one laboratory scientist and one veterinarian. In keeping with the “One health” concept, to mitigate the increasing threat of outbreaks of zoonotic diseases and to further strengthen the laboratory's key role in public health surveillance and response in the country, the trainees/residents were selected from serving staff nominated by the Ghana Health Service, Ministry of Health (physicians and laboratory scientists) and the Veterinary Service Directorate, Ministry of Food and Agriculture (veterinarians).

The vision of GFELTP is to improve the health of the people in Ghana. The mission is to contribute to addressing Ghana's public health needs and priorities through training and service provision in applied epidemiology and public health laboratory management.

The objectives of GFELTP are to: 1) Strengthen public health capacity by developing a cadre of health professionals with applied skills in applied epidemiology and laboratory management; 2) Contribute to research activities on priority public health problems; 3) Improve national capacity to respond to public health emergencies such as disease outbreaks, natural disasters and unusual public health events including those that could be a result of chemical or bioterrorism; 4) Strengthen national surveillance systems through a team approach (physicians, laboratory scientists and veterinarians); 5) Improve communications and networking of public health practitioners in the country and throughout the Africa Region.

### Brief outline of the course

The GFFELTP is a 2 calendar-year programme with about 30% course work and 70% field work covering two tracks (i.e., the epidemiology track and the laboratory track). During the first academic year, residents study core courses that cut across the two tracks in the first semester. In the second semester, residents take courses in each of the prescribed track (i.e., epidemiology for medical and veterinary professionals or laboratory for laboratory scientists) and some selected electives to make up for the required 36 credits for the course work. In addition, residents are required to be involved in 16 weeks of field activities made up of 8 weeks at the end of the first semester to undertake evaluation of surveillance systems of selected diseases and 8 weeks at the end of the second semester for analysis of available large datasets on diseases at national or regional levels. In the second year, residents develop their research topics under the guidance of their academic supervisors and mentors. A further requirement is the organization of at least one seminar prior to going for the field work. Ten months of the second year are devoted to field practice and collection of data while providing services to the district/region of assignment. The last two months are used for data analysis and write up of theses. During the 2-year period of training especially when on field postings, residents of the programme join the staff of the Ghana Health Service and Veterinary Service Directorate to investigate and respond to disease outbreaks and public health emergencies. Being mid-career professionals in public service, the residents sometimes lead these investigations, conduct public health interventions and present written and verbal reports to stakeholders with support of their supervisors and mentors.

### Residents

**Figure 1:**
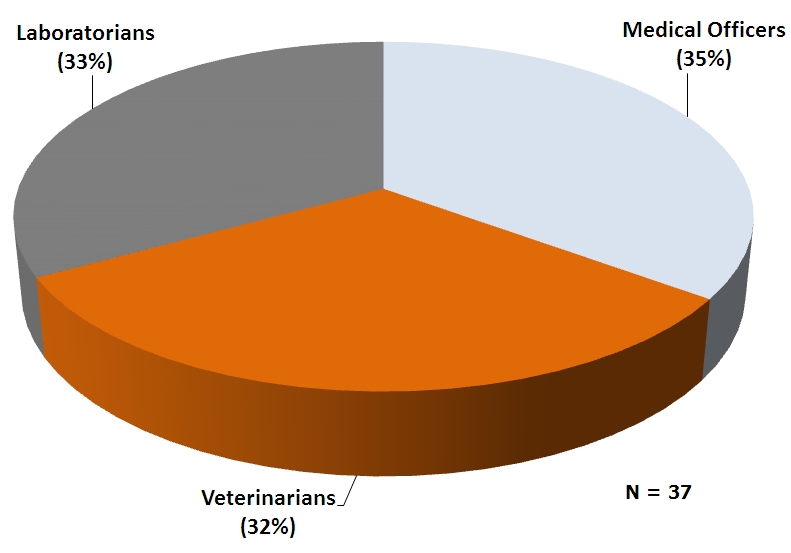
Proportion of Ghana Field Epidemiology and Laboratory Training Program residents by profession

**Figure 2:**
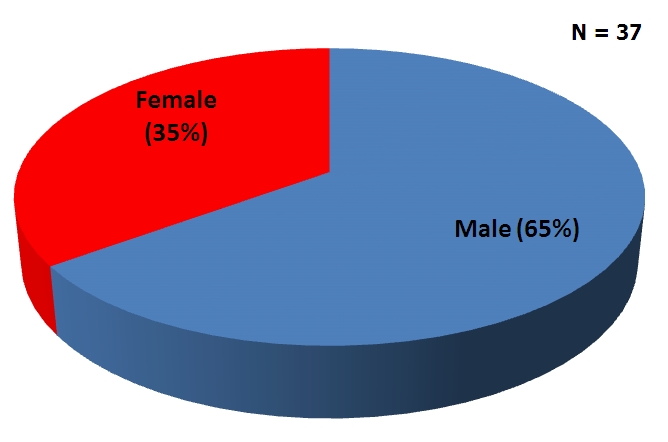
Sex distribution of Ghana Field Epidemiology and Laboratory Training Program residents

Five cohorts have so far been admitted into the residency programme. The breakdown is as shown in Table 1. The distribution of residents by professional background and sex is shown in Figure 1 and Figure 2.

**Table 1:** The Ghana Field Epidemiology and Laboratory Training Program (GFELTP) residents enrolment by track and training period

	Number of Residents	Epidemiology Track	Laboratory Track
Cohort 1 (2007-2009)	5	4 (3 physicians, 1 Veterinarian)	1
Cohort 2 (2008-2010)	8	5 (2 physicians, 3 Veterinarians)	3
Cohort 3 (2009-2011)	7	5 (2 physicians, 3 Veterinarians)	2
Cohort 4 (2010-2012)	8	5 (3 physician, 2 Veterinarians)	3
Cohort 5 (2011-2013	9	6 (3 physician, 3 Veterinarian)	3
**Total**	**37**	**25**	**12**

## Major achievements of the program

### Pre-GFELTP Activities

Workshop on Outbreak Investigations for Disease Control Officers, Kintampo Rural Health Training School (May 2003). Graduates of the Training School serve as disease control officers responsible for surveillance and outbreak investigation activities at the district and facility levels of the health system. The workshop was organized to further train the trainee-officers on principles of disease outbreak investigation using modules adapted from the US CDC FELTP training materials.

### 1) Needs assessment in five selected districts on Integrated Disease Surveillance and Response (IDSR), 16 April – 7 May, 2005

As part of the collaboration between the Disease Surveillance Department(DSD) and the School of Public Health (SPH), a needs assessment to determine the gaps in disease surveillance with emphasis on disease outbreak investigations and response, data analysis and interpretation and capacity development was conducted in fivedistricts in Ghana, namely Asuogyaman, Ketu, Kassena-Nankana, Wassa West and Berekum Districts. Needs assessment tools were developed and discussed by DSD and SPH at an orientation before the exercise commenced. The reports from these assessments were compiled into a composite document for implementation of sensitization workshops for the districts.

### 2) Workshop on Disease Outbreaks, Investigations and Response in Asuogyaman District, 11-15 July 2005

The workshop had 19 participants, made up of Disease Control Officers, Nurses, Statistician, Midwives, and Medical Superintendent at the Volta River Authority (VRA) Hospital, Medical Assistant and District Director of Health Services. The general objective of the workshop was to give health workers in the district the appropriate knowledge and skills in identifying cases of priority diseases and also process the data and use it for public health action. In addition, core stakeholders such as district assembly members, immigration and custom officers, teachers, information officers, the police and the media were also involved.

The specific objectives were to enable participants to: detect priority diseases, analyze and interpret data on priority diseases, investigate and respond to suspected outbreaks, be prepared for disease epidemics, investigate and respond to other priority diseases, supervise and provide feedback and be able to monitor and evaluate IDSR implementation.

The workshop employed methods including presentations on Integrated Disease Surveillance and Response training modules, role-playing, group work and field exercises. Similar workshops were organized in Ketu, Upper East and Berekum districts. As a result of these workshops, participating districts reported improvements in their disease detection, investigation and public health response.

Major activities undertaken by GFELTP residents over the years are summarized as follows:

**Disease outbreak investigations:** A total of 23 disease outbreak investigations were conducted by GFELTP residents between 2007 and 2011. These include outbreaks on meningitis, influenza (type A), human rabies, food borne diseases, measles, gastrointestinal diseases, Yellow fever, pertussis, cholera and herpes B. The investigation of an outbreak of herpes B virus infection in May 2011 in Techiman and adjoining districts of central Ghana reported this virus as the probable cause of zoonotic encephalitis in Ghana for the first time.The large number of disease outbreak investigations and the timely response that residents of the programme have been able to carry out alongside other Ghana Health Service or Veterinary Service staff to date have appreciably enhanced disease surveillance and response capacity in the country. In particular, the role that GFELTP team of physicians, veterinarians and laboratory scientists played in the investigation and response to the AI outbreak in Ghana in 2007, the multiple outbreaks of rabies in 2009 -2011, and the monkey-associated herpes-B encephalitis outbreak in 2011 demonstrated the great value of the One Health concept and the multi-disciplinary team approach which the GFELTP has adopted.

**Disease surveillance and field studies:** As part of end-of-year one field requirements, 31 evaluations of various disease surveillance systems were conducted between 2008 and 2011. They included both communicable and non-communicable diseases. Residents have also analyzed available large datasets for 28 selected diseases at the regional health directorates.

### 3) Scientific conferences

### TEPHINET conferences

Brasilia, Brazil, December, 2006: Six residents presented abstracts at the 4^th^ TEPHINET Scientific Conference. The presentations were made up of two oral presentations and four poster presentations.

Kuala Lumpur, Malaysia (November 2008): During the Fifth TEPHINET Scientific Conference in Kuala Lumpur, Malaysia in November 2008, GFELTP residents presented six abstracts, made up of two orals and four posters.

Cape Town, South Africa (December, 2010): At the 6^th^ TEPHINET Global Scientific Conference, Nine GFELTP residents presented three orals and six poster presentations. One of them, Ms. Joyce Der, a cohort-II laboratory track resident was the overall winner in the oral presentation category. She presented the epidemiological and laboratory investigation of a food poisoning outbreak at a popular urban-area food center in the Eastern Region of Ghana.

### African Regional Conferences

Accra- Ghana, December, 2005: GFELTP hosted the 1^st^ AFENET Regional Scientific Conference following the birth of AFENET in August of the same year in Accra Ghana [4]. The residents made five oral presentations and six poster presentations.

Kampala, Uganda- December 2007: Nine presentations were made by GFELTP residents at the 2^nd^ AFENET Regional Scientific Conference in Kampala. Four were orals and five were poster presentations.

Mombasa, Kenya- August 2009: A total of 15 posters and 6 oral presentations were made at the 5^th^ TEPHINET African Regional/3^rd^ AFENET Scientific Conference by GFELTP residents. One of them, Dr Paul Polkuu, a veterinarian and cohort II epidemiology track resident received the runner-up award for the best poster presentation. The presentation was on the investigation of an Influenza-like Illness (ILI) outbreak at a co-educational high school in the Eastern Regional mountains of Ghana.

### Epidemic Intelligence Service (EIS) Conferences, Atlanta

Two GFELTP residents made presentations at the EIS Conference International Night meetings in 2010 and 2011. The respective presentations were titled; Cross border Rabies Outbreak, North-Eastern Ghana, and Cholera Outbreak in East Akim District, Ghana.

### International Epidemiological Association (IEA) World Congress of Epidemiology

One resident won the International Epidemiological Association bursary award and made a poster presentation titled; Progress Towards Eradication of Poliomyelitis in Ghana: A Review of the Eastern Region at the 19^th^ Annual World Conference in Edinburgh, Scotland in August 2011.

Two other residents had three papers accepted for presentation at the 2011 International Society of Infectious Diseases conference on Neglected Tropical Diseases held in Boston, USA but could not attend due to funding limitations.

### 4) Residents publications

A paper by a cohort-II epidemiology track resident “Community-wide outbreak of cholera following unhygienic practices by small-scale unregistered gold miners, East-Akim District, Ghana – 2010” was accepted for publication by the Ghana Medical Journal in September, 2011 Four public health articles by residents have been published in two veterinary bulletins and two national daily newspaper columns

### 5) Onsite field supervision mentorship and public health advocacy

In addition to SPH faculty members, selected Regional Directors of Health Services and District Directors of Health Services were oriented from the start of GFELTP to serve as supervisors and mentors for residents at various field sites. In May 2009, a Resident Advisor was appointed for GFELTP. Since then, in collaboration with the Ghana Health Service Public Health Division, he has conducted periodic rounds of visits to residents’ Field Sites. The aims of the visits are to 1).provide mentorship, supervision and tutoring to residents during their field trainings, 2) conduct local stakeholders’ feedback and public health consensus seminars and 3) conduct program advocacy and sensitization meetings with key stakeholders at regional and district levels. Multiple visits have been made to the Eastern, Central, Brong-Ahafo, Greater Accra Northern, Upper West, Upper East, Volta and Western regions. There have been 10 regional stakeholders’ seminars where residents made presentations on projects they undertook in various regions or districts to stakeholders from the community, Ghana Health Service and Veterinary Services Directorate. These fora provided opportunities for feedback, inter-sectoral discussions leading to consensus on public health action and sharing of information on GFELTP activities and opportunities. This novel approach of collaborative training and service at the local level has enhanced public health decision, action and GFELTP visibility at the health system frontline level.

### 6) Improving Management of Public Health Interventions Workshops

The GFELTP has hosted three workshops on Improving Management of Public Health Interventions. This followed an introductory course to train proposed trainers in 2008. The trainers were Deputy Directors in charge of Public Health at Regional level in Ghana. There were 17 participants and the training was facilitated by CDC, Ghana Health Service (GHS) and GFELTP staff. The first workshop was held from June 22- July 17, 2009 and was targeted at health practitioners in the African Sub-Region. Twenty-two health officials from four African Countries attended the course. Out of the 22 participants, 19 were Ghanaians, 1Kenyan, 1 Tanzanian and 1 Ugandan. All 19 Ghanaian participants were staff from the Ghana Health Service. The course was divided into four modules. These four modules were designed to touch on all aspects of health management. The faculty was made up of sixteen staff from University of Ghana Business School (UGBS), Ghana Institute for Management and Public Administration, AFENET, SPH, and CDC- SMDP.

Modules covered were: 1) Leadership, Networking and Advocacy; 2) Project Planning and Management for Public Health; 3) Operational Management; 4) Monitoring and Evaluation for Health Programs

The second workshop was held from 21st June to 16th July 2010 and had a cohort of 16 trainees from Ghana (11), Nigeria (4) and Rwanda (1). Participants from Ghana were staff of Ghana Health Services made up of District Medical Directors and those from Nigeria were made up of lecturers in University, FELTP residents and staff of CDC.

Uniqueness of the course was that during the four-week period, participants presented project proposals on management of public health interventions at the beginning of the course. They were helped to develop the proposals and implement them over the subsequent three months after the course. All participants were visited by a facilitator once during the three months of implementation. The Ghanaian participants came back for a day to present the results of what they implemented before receiving their certificates. The regional participants were visited by the coordinator and the AFENET focal person for the course in their various countries. Participants made their presentations at a meeting of stakeholders before they were awarded their certificates.

The workshop with the field component was evaluated six months after the first four-week IMPHI course ended. The goal of the evaluation was to determine whether the four-week training led to application of skills on the job as outlined in the curriculum and program objectives. It was a joint evaluation by CDC-SMDP and stakeholders at the School of Public Health in Ghana.

Six months after the 4-week IMPHI course ended all 12 participants who were interviewed for this evaluation reported implementing a change in management practice at their places of work. Only one participant interviewed could not provide any hard evidence for any of the changes she implemented.

The 2011 Edition of IMPHI course was held from August 1 to August 12, 2011. There were 21 participants from Cohorts II, III, and IV Residents of the GFELTP. Topics treated at the Workshop were: Introduction to Leadership, Vision, Mission; Personal & Organizational Development; Grant Proposals Writing; Report Writing; Principles of Monitoring & Evaluation Frameworks; Monitoring & Evaluating a Public Health Programme; Intervention Evaluation Methods. All participants were issued with certificates.

### 7) Integrated Avian Influenza outbreak and pandemic influenza course

In collaboration with the USAID/STOP AI Programme, the GFELTP, in May 2010, pre-tested a newly developed set of modules on Integrated Avian Influenza Outbreak Response and Pandemic Influenza in a special two-week training workshop. The purpose of the workshop was to determine the usefulness of these modules in the African setting, with a view of introducing these modules in other FELTPs. The GFELTP has since then adapted materials from the modules into the GFELTP curriculum, and it has been organized yearly with facilitators from the Veterinary Services, School of Veterinary Medicine, National Disaster Management Organization (NADMO) and SPH.

### GFELTP graduates strengthening public health workforce in Ghana

GFELTP collaboration with the Veterinary Services Directorate, Ministry of Food and Agriculture in Ghana has led to the strengthening of the regional epidemiology capacity of the service. Two GFELTP graduates currently serve as the regional veterinary epidemiologists in the Brong-Ahafo and Upper West regions. Two others are awaiting appointment letters to serve as regional epidemiologists in the Central and Volta regions. Similarly, the Ghana Health Service is finalizing formal plans to deploy the GFELTP graduates to fill such positions in the regions. Currently, two of the graduates serve as deputy national program managers for malaria and non-communicable diseases respectively, one as deputy head of the national public health and reference laboratory, three as district directors of health service and de facto regional epidemiologists (Table 2).

**Table 2:** The placement of the Ghana Field Epidemiology and Laboratory Training Program (GFELTP) graduates pre and post certification

COHORT 1		
Resident	Position before training	Position after training
**1**.	Medical Superintendent	Deputy Programme Manager (Cancer Programme-NCD Programme)
**2**.	Public Health Physician	Deputy Programme Manager (National Malaria Control Programme)
**3**.	District Director (Veterinary Services, Eastern Region)	Regional Epidemiologist (Veterinary Services, BrongAhafo Region)
**4**.	Biomedical Scientist (National Public Health & Reference Lab- GHS)	Deputy Head (National Public Health & Reference Lab-GHS)
**5**.	Medical Superintendent	District Director of Health Services West Gonja District, Northern Region
**COHORT 2**		
**6**.	Principal Veterinary Officer (MOFA) Jirapa District, UWR	Deputy Director and Regional Veterinary Epidemiologist (MOFA, Upper West Region)
**7**.	Senior Veterinary Officer, (Greater Accra)	Principal Veterinary Officer (Greater Accra)
**8**.	Biomedical Laboratory Scientist (Public Health Reference Lab.)	Biomedical Laboratory Scientist (Public Health Reference Lab.)
**9**.	Biomedical Laboratory Scientist (Korle-Bu Teaching Hospital, Central Lab.)	Biomedical Laboratory Scientist (Korle-Bu Teaching Hospital, Central Lab.)
**10**.	Biomedical Scientist (Regional Hospital, Koforidua)	Biomedical Scientist (Regional Hospital, Koforidua)
**11**.	District Director of Health Service, GHS-Dormaa.	Municipal Director of Health Services,GHS-Dormaa.
**12**.	Municipal Veterinary Officer, AkimOda	Municipal Veterinary Officer, AkimOda
**13**.	District Director, Health Services, Akwapim South-Atibie	District Director of Health Services Akwapim North District, Eastern Region

### GFELTP Steering Committee

A steering committee made up of representatives from stakeholders (MOH, GHS, Veterinary Services, Laboratory Services, NADMO, CDC, Noguchi Medical Research Institute and SPH) steers the management of the GFELTP to achieve the objectives of the programme. The Committee is chaired by MOH/GHS and it meets every quarter. The meetings are well documented and shared with all members/partners. The committee follows up plans and recommendations through designated members with support of GFELTP secretariat.

### GFELTP assessment

A Matrix Tool for FELTP Assessment was used to do an internal evaluation of the programme and the result presented to the GFELTP Steering Committee. The Ghana FELTP has also gone through assessment by AFENET and awarded Quality Assurance Certificate for 2010

### Placement of Graduates

Table 2 shows the placement of graduates, pre and post certification.

## Discussion

The genesis and evolution of GFELTP is an example of a national identification of a workforce capacity need and the use of multi-sectoral collaboration with international technical and financial assistance to institutionalize indigenous capacity development in applied epidemiology. The SPH at the University of Ghana is a well-established constituent member of the College of Health Sciences of the University. The MPH program which is the flagship program of the School is thriving well with enrolment from Ghana, the African Region and beyond. The GFELTP was developed as a special program based in the Epidemiology Department of the School. The contribution of the FELTP to the strengthening of the epidemiology curriculum of the MPH program in the SPH has been acknowledged by both graduates and the Ministry of Health at several of the School's annual dissemination forum. The current policy of the Ministry of Health and the Veterinary Services Directorate of deploying graduates of the GFELTP in strategic posts in the national public health service clearly shows the appreciation of the competencies and skills of the graduates.

The outputs of the residents of the GFELTP have demonstrated the scientific rigor that has characterized the field investigations and dissertations that have been produced. Two of the members of the initial cohort have submitted their upgraded dissertations for the award of PhD in epidemiology as of 2011. The emphasis on scientific writing and communication has also reflected in the oral and poster presentations that residents from the program have made in Regional and Global Scientific Conferences. The graduates of the program have all returned to positions with an evolving career structure that is likely to motivate them to remain in the public health service. As part of the new public health institute model facilitated by the international association of public health institutes (IANPHI) initiative in Ghana, the Ghana Health Service is developing a core public health technical or expert team career path that uses the GFELTP graduates to fill the critical role of epidemiologists at the subnational and national levels as well as along specific disease control or public health program lines. Crossover to public health administration track at the top of the path is an option and defined promotion track in keeping with the national public health service policy has been proposed. There is ample evidence of improved public health surveillance and response as well as evidence-based decision making taking place in the National Health Service following the joint evaluation of surveillance systems, disease dataset analyses, outbreak investigations, public health interventions with more regular reports, information sharing and periodic stakeholders’ public health seminars at all levels. There has been a definite strengthening of the public health workforce and increased networking between programs in Ghana and with other countries [5]. The prospect of increasing support from the local stakeholders should see increasing enrolment in the program as demonstrated by the 2011 enrollment of nine service professionals, the highest number so far of the five cohorts. This should hasten the attainment of the vision and mission of the program.

## Challenges

The major challenge of the GFELTP has been the slow follow up on pledges of the major national stakeholders of the programme in honouring their funding commitments as specified in the Memorandum of Understanding (MOU). This has resulted in limiting the number of qualified residents that could have been admitted into the programme. But from testimonies that all stakeholders have given on various occasions about the value they place on the service provided by graduates of the programme, it is expected that their support should be forthcoming. At the formal public launching and 1^st^ certification ceremony of the programme on 02 June 2011, the Minister of Health and the Director of Veterinary Services both emphasized their new policy of utilizing the graduates of the GFELTP in strategic positions in the public health system of the country in order to improve the response to existing public health threats and the emerging zoonotic diseases. These pronouncements encourage our optimistic view regarding the programme sustainability based on continuing support from these key indigenous stakeholders.

## Prospects for the future

There is no doubt that the establishment of the SPH and the subsequent addition of the GFELTP in Ghana has contributed significantly to addressing the competency and skills needs of the public health workforce. This is evidenced by the large number of disease outbreak investigations and the timely response that residents of the programme have been able to carry out to date. In particular, the role that GFELTP team of physicians, veterinarians and laboratory scientists played in the investigation and response to the AI outbreak in Ghana in 2007 demonstrated the great value of the One Health concept and the team approach, which the GFELTP has adopted. The unique feature of the GFELTP that permits trainees to provide service to the Public Health Service even while still in training has made the outputs of the trainees well appreciated by relevant employers. Consequently, the demand for the course has been growing. As more local stakeholders’ support come on board, it is expected that larger numbers of trainees will be admitted into the programme in order to respond to increasing challenges of growing complex of public health emergencies in the country and the Sub-region.
